# Cost-effectiveness analysis of Ado-trastuzumab emtansine for the treatment of residual invasive HER2-positive breast cancer

**DOI:** 10.31744/einstein_journal/2022GS6655

**Published:** 2022-05-02

**Authors:** Marcos Aurélio Fonseca Magalhães, Pedro Nazareth Aguiar, Milena Brachmans Mascarenhas Neves, Gilberto de Lima Lopes, Auro del Giglio

**Affiliations:** 1 Centro Universitário FMABC Santo André SP Brazil Centro Universitário FMABC, Santo André, SP, Brazil.; 2 Sylvester Comprehensive Cancer Center Miami FL United States Sylvester Comprehensive Cancer Center, Miami, FL, United States.

**Keywords:** Cost-benefit analysis, HER2-positive, Breast neoplasms, Ado-trastuzumab emtansine, Neoplasm, residual

## Abstract

**Objective:**

Human epidermal growth factor receptor 2 (HER2) overexpression occurs in up to 30% of breast cancer cases. Ado-trastuzumab emtansine (T-DM1) is approved to treat residual HER2-positive breast cancer after neoadjuvant therapy. The aim of this study was to determine the quality-adjusted time with symptoms or toxicity and without symptoms or toxicity (Q-TWiST) of T-DM1 compared to trastuzumab for residual invasive HER2-positive breast cancer.

**Methods:**

The authors developed an analytical model extracting individual patient data and estimated invasive disease-free survival and overall survival over a 30-year time horizon. Only direct costs from adjuvant treatment were considered as well as relapse treatment from Brazilian and American payer perspectives. Heart events were considered for utility and cost analysis.

**Results:**

The 30-year projection utilizing the Weibull method estimated a mean invasive disease-free survival of 16.4 years for T-DM1 and 10.4 for Trastuzumab, in addition to a mean overall survival of 18.1 and 15.4 years, respectively. We determined a Q-TWiST gain of 3,812 years for the T-DM1 arm when compared to trastuzumab and an Incremental cost-effectiveness ratio per Q-TWiST of US$ 11,467.65 in the United States and US$ 3,332.73 in Brazil.

**Conclusion:**

Ado-trastuzumab emtansine is cost-effective from both Brazilian and American perspectives.

## INTRODUCTION

Breast cancer is the most diagnosed cancer, and it has the highest mortality in women in more than 100 countries worldwide, with estimates from 2018 registering over 2.1 million new cases, 626,679 deaths, and representing 11.6% of all cancer deaths combined in the world.^( 1 , 2 )^

Amplification or hyperexpression of the human epidermal growth factor receptor 2 (HER2), present in 15% or more of invasive breast tumors,^( 3 )^ is a predictor of both overall survival and disease-free survival (DFS). Trastuzumab binds to the HER2 extracellular domain and prevents the activation of intracellular tyrosine kinase,^( 4 )^ in addition to recruiting immune effector cells that are responsible for antibody-dependent cytotoxicity.^( 5 )^

After proven benefit for patients with advanced HER2-positive breast cancer, anti-HER2 therapies were evaluated for early disease.^( 6 , 7 )^ In 2005, Piccart-Gebhart et al. evaluated treatment with one to two years of trastuzumab in HER2- positive early breast cancer with prior neoadjuvant or adjuvant therapy, showing an overall survival benefit of one year against the observational group.^( 8 )^

The use of anti-HER2 therapy in the neoadjuvant setting resulted in an important increase in the pathological complete response rate (pCR),^( 9 )^ defined as the absence of residual cancer in the breast and axillary nodes.^( 10 )^ The results from the TECHNO trial showed a 3-year increase in DFS and overall survival of 15 and 10%, respectively, in patients who achieved pCR compared to those who did not.^( 11 )^ As a result, the treatment of patients with residual disease after neoadjuvant therapy remain a challenge.

Ado-trastuzumab emtansine (T-DM1) is a drug composed of the association of trastuzumab with the cytotoxic agent emtansine (DM1), inducing cell death through microtubular inhibition.^( 12 )^ In 2018, von Minckwitz et al.^( 13 )^ evaluated the use of adjuvant T-DM1 in patients with residual disease after neoadjuvant treatment with taxane and trastuzumab in the KATHERINE trial, indicating a 50% reduction in the risk of relapse in the T-DM1 group.

Developing new treatments for cancer that increase survival is the main goal in clinical trials, but not less important is to evaluate the impact in quality of life (QoL) with each new therapy. In addition, the cost of such new treatments is also important. The cost of treating cancer has been increasing over the years. Worldwide, the annual cost is approximately US$ 100 billion, and it is expected that in 2020 the cost may reach up to US$ 150 billion.^( 14 , 15 )^ In the United States of America (USA) alone, the average price of a new cancer drug exceeds US$ 100 thousand annually. New drugs and treatment technologies for common diseases such as breast cancer can be unaffordable to people in lower- and middle-income countries,^( 16 )^ exceeding household incomes^( 14 )^ even if they are considered cost-effective.^( 17 )^

The efforts to integrate both QoL and quantity of life led to development of the adjusted quality of time without symptoms and toxicity (Q-TWiST),^( 18 )^ that evaluates the survival time that remains after subtracting periods of time with symptoms of disease and toxicity from the overall survival time.^( 18 )^ It is a measure of quality of survival that correlates the time without and with symptoms, and the treatment cost.^( 19 , 20 )^

## OBJECTIVE

To evaluate the quality-adjusted time with and without symptoms or toxicity of ado-trastuzumab emtansine in the adjuvant setting compared to trastuzumab for residual invasive HER2-positive breast cancer.

## METHODS

**Figure 1:**
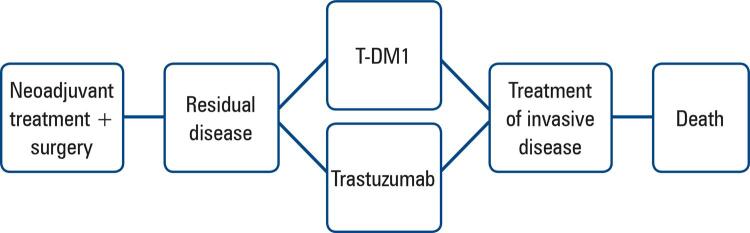
Decision-analytic model

We developed an analytical model to assess the cost-effectiveness of T-DM1 *versus* trastuzumab for residual invasive HER2-positive breast cancer treatment ( Figure 1 ).

We obtained the effectiveness of the proposed treatments using data from the KATHERINE study and analyzed the data using costs in the USA and the Brazilian private medical systems. This study considered costs of drugs and adverse events during and after treatment in addition to end-of-life costs.^( 21 - 23 )^ We based the price of T-DM1 and trastuzumab complete treatment on information taken from UpToDate (USA)^( 24 )^ and *Brasíndice* (CMED Brazil).^( 25 )^ A hypothetical 70kg patient was considered as the basis for the doses, setting four cycles of neoadjuvant trastuzumab with a loading dose of 8mg/kg and a maintenance dose of 6mg/kg every 3 weeks, followed by 14 cycles of trastuzumab 6mg/kg *versus* 14 cycles of T-DM1 with a dose of 3.6mg/kg.

Q-TWiST is determined as the sum of the quality-adjusted (u) time spent undergoing treatment and experiencing toxicity of any grade (TOX), plus the time spent free of disease in perfect health (TWiST), plus the time spent experiencing symptoms in disease relapse (REL). To calculate the TOX value, we evaluated the heart failure treatment cost during treatment with T-DM1 and trastuzumab. To estimate REL it was subtracted the overall survival from the invasive disease-free survival (IDFS), generating a value determined as post progression survival (PPS).^( 20 )^


$$Q-TWiST=u_{\text{tox }}\times TOX+u_{TWiST}\times TWiST+u_{REL}\times REL$$

To determine the incremental cost-effectiveness ratio (ICER), we subtracted the total cost values, considering the costs of adverse effects and post progression for T-DM1 (C_1_) and trastuzumab (C_0_) and dividing them by the difference of the Q-TWiSTs calculated for each medication (E_1_ and E_0_).^( 26 )^


$$ICER=\frac{C_{1}-C_{0}}{E_{1}-E_{0}}$$

The Brazilian currency (real) was converted into USA dollars to facilitate standardization using the rate of 4.05 Brazilian reais for each USA US$ 1.00.

The primary endpoint of the study was ICER expressed by the incremental cost to add one year of overall survival without symptoms.

### Model structure

In this analytical decision model, we considered the survival time: in adjuvant treatment, IDFS, relapse and death. All of them were adjusted with the respective utilities.

### Clinical effectiveness and quality of life

Invasive disease-free survival data and overall survival in adjuvant treatment with T-DM1 and trastuzumab were taken from the KATHERINE study with extraction of individual data following the method of Guyot et al.^( 27 )^ and Kaplan-Meier graphics were created with WebPlotDigitizer.

A Weibull distribution was performed using a 30-year time projection from the final available follow-up data for IDFS and overall survival using a non-parametric Kaplan-Meier survival estimator from the data published in the KATHERINE study. The utilities for progression-free survival (PFS) and PPS were calculated from data already published.^( 21 - 23 )^

### Medical costs

In addition to the costs for purchasing drugs, the cost for post progression were considered according to data already published.^( 21 - 23 )^ In terms of cost of adverse events, we considered only heart failure costs,^( 23 )^ due to being the most important adverse events related to Trastuzumab and T-DM1. To calculate costs from the Brazilian perspective, we used the Power Purchasing Parity (PPP) of 2018, determined by the World Bank as 2.02.^( 28 )^

### Deterministic sensitivity analysis

Several unidirectional deterministic sensitivity analyses were performed to assess the influence of uncertainty on individual Q-TWiST calculations. We have included a 95% confidence interval (95%CI) for the most important variables. To determine the probability of T-DM1 cost-effectiveness, we used a threshold of purchase of US$ 30,000 for Brazil and US$ 180,000 for the USA, calculated from the multiplication of the gross domestic product per capita (GDP) of each country, following World Health Organization (WHO) criteria.^( 29 )^

## RESULTS

**Figure 2:**
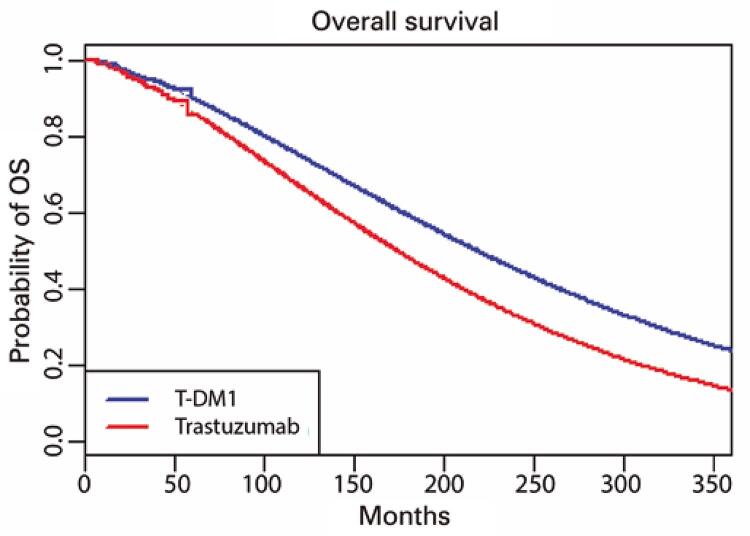
Kaplan-Meier with Weibull overall survival

**Figure 3:**
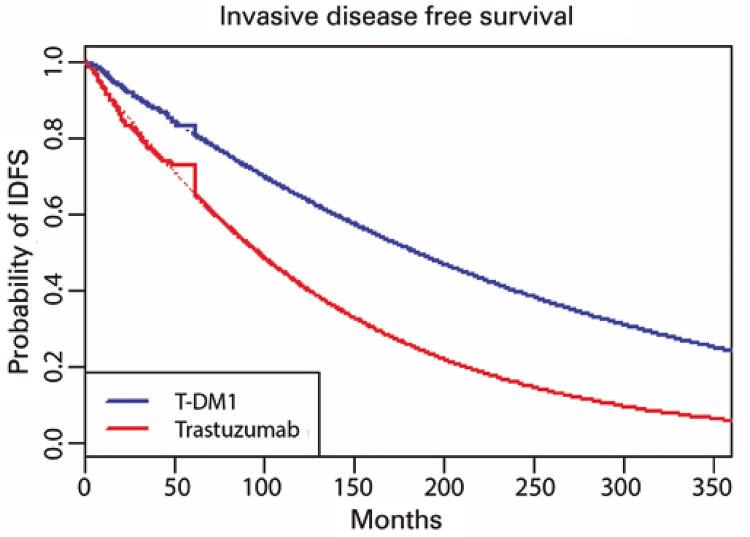
Kaplan-Meier with Weibull invasive disease-free survival

Applying the Weibull distribution with the corresponding 30-year setting, the IDFS was 16.4 years for T-DM1 and 10.4 years for trastuzumab, with an overall survival of 18.1 and 15.4 years, respectively ( Figure 2 and Figure 3 ). The Q-TWiST values were 17.310 and 13.459 for T-DM1 and trastuzumab, respectively. We observed an ICER per Q-TWiST of US$ 11,467.65 in the USA and US$ 3,332.73 in Brazil ( Table 1 ).

OS: overall survival.

IDFS: invasive disease-free survival.

**Table 1:** Summary of base-case analysis

Parameters	USA		Brazil	
	T-DM1	Trastuzumab	T-DM1	Trastuzumab
Number of cycles^(13)^	14	14	14	14
Cost per cycle^(24,25)^	US$ 11,129.28	US$ 5,714.19	US$ 5,213.34	US$ 3,170.79
Total drug treatment cost	US$ 178,666.76	US$ 102,855.42	US$ 85,669.92	US$ 57,074.22
Cost per event after progression^(20)^	US$ 322,178		US$ 159,494.05	
Post progression cost	US$ 39,459.22	US$ 71,456.93	US$ 19,534.26	US$ 35,419.27
Cost per adverse event (heart failure)^(23)^	US$ 4,458.50		US$ 2,207.17	
Adverse events cost	US$ 54.01	US$ 60.01	US$ 26.73	US$ 29.7
Total costs	US$ 218,179.98	US$ 174,462.36	US$ 105,230.91	US$ 92,523.19
Utilities^(21-23)^	uTOX 0.64 uADJ 0.97 uTWiST 0.99			
Mean IDFS (years)	16.4	10.4	16.4	10.4
Mean PPS (years)	1.7	5	1.7	5
Mean OS (years)	18.1	15.4	18.1	15.4
Q-TWiST (years)	17.310	13.459	17.310	13.459
T-DM1 ICER per Q-TWiST	US$ 11,467.65	-	US$ 3,332.73	-

T: trastuzumab; PFS: progression-free survival; PPS: post-progression survival; OS: overall survival; Q-TWiST: quality-adjusted time without symptoms or toxicity; ICER: incremental cost-effectiveness ratio; T-DM1: Ado-trastuzumab emtansine; IDFS: invasive disease-free survival.

**Figure 4:**
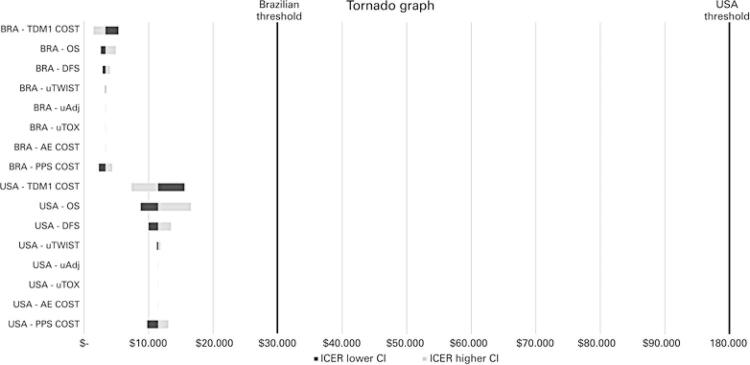
Deterministic sensitivity analysis

BRA: Brazil; OS: overall survival; DFS: disease-free survival; TWiST: time spent free of disease in perfect health.

In the USA, the total cost of treatment with T-DM1 is US$ 154,355.46 and with trastuzumab is US$ 102,855.50. In Brazil the costs are US$ 72,179.64 and US$ 57,074.22, respectively ( Table 1 ). Drug prices had the most influence on cost, followed by treatment and PPS. Due to low cost per event, adverse events had little impact in the final price ( Table 2 and Figure 4 ).

**Table 2:** Deterministic sensitivity analysis parameters

Parameters	Mean deterministic	95%CI	
		Lowest value	Highest value
Costs			
Post-progression (per event)	US$ 322,178 (USA) US$ 159,494.05 (BRA)	US$ 261,309.20 (USA) US$ 127,595.24 (BRA)	US$ 386,613.60 (USA) US$ 191,392.86 (BRA)
Heart failure cost (per event)	US$ 4,458.50 (USA) US$ 2,207.17 (BRA)	US$ 3,566.80 (USA) US$ 1,765.73 (BRA)	US$ 5,350.20 (USA) US$ 2,648.60 (BRA)
T-DM1 cost (per cycle)	US$ 11,129.28 (USA) US$ 5,213.34 (BRA)	US$ 10.016,35 (USA) US$ 4,692.00 (BRA)	US$ 12,242.20 (USA) US$ 5,734.67 (BRA)
Trastuzumab cost (per cycle)	US$ 5,714.19 (USA) US$ 3,170.79 (BRA)	US$ 5,142.77 (USA) US$ 2,853.71 (BRA)	US$ 6,285.60 (USA) US$ 3,487.86 (BRA)
Outcomes			
Adjuvant Toxicity Utility (uTOX)	0.64	0.62	0.66
Adjuvant Utility (uADJ)	0.97	0.95	0.99
Adjuvant TWiST Utility (uTWiST)	0.99	0.97	1.00

95%CI: 95% confidence interval; BRA: Brazil; TWiST: time spent free of disease in perfect health.

The drug’s price was the factor that most influenced the final costs when analyzing the Deterministic Sensibility Analysis (DSA) in the USA and Brazil. Trastuzumab and T-DM1 costs represented 58% and 81% of the total cost in the USA, respectively; and 61% and 58% of the total cost in Brazil, respectively.

Considering the base ICER value of US$ 11,467.63 to USA and US$ 3,332.73 to Brazil, a decrease of 10% in T-DM1’s price resulted in a total reduction of ICER value to US$ 7,379 in the USA and a reduction to US$ 1,497 in Brazil ( Figure 4 ).

## DISCUSSION

In an analysis published in 2016,^( 30 )^ the cost of treatment with T-DM1 in the USA for second-line treatment for HER2-positive metastatic breast cancer was evaluated using the combination of trastuzumab, pertuzumab, and docetaxel (THP) as the first line and lapatinib with capecitabine as the third-line treatments. Using the Markov model, a quality-adjusted life year (QALY) of 1.81 was determined at a cost of US$ 335,231.35 and suggested that, to be cost-effective, there should be a 50% reduction in the total price of the drugs. In a similar analysis,^( 31 )^ but this time using treatment in Taiwan as scenario, it was observed that in the first-line treatment with only trastuzumab and docetaxel (TH), following the same configuration for the second- (T-DM1) and third-line treatments (capecitabine and lapatinib), it would still not be cost-effective.

The absence of a favorable cost-effectiveness ratio in the advanced disease setting emphasizes the importance of cost-effectiveness studies to find the patients who will benefit most from such therapies and how to apply limited resources in healthcare.

In addition to our results, two other analyses demonstrated positive results for the use of T-DM1 in the adjuvant setting in the USA, showing reduced cost for treatment and lesser toxicity compared to more intensive therapy.^( 32 , 33 )^ In our study we found that T-DM1 is cost-effective not only in the USA perspective but also in the Brazilian perspective.

Regarding the cost-effectiveness analyzes of other regimens for early disease, Garrison et al. published a cost-effectiveness analysis comparing pertuzumab, trastuzumab, and docetaxel (PHT) *versus* pertuzumab and trastuzumab (HT) in the adjuvant setting; this showed an ICER of US$ 167,185 per QALY gained (0.45 QALY), defined as cost-effective in patients with high-risk node-positive disease.^( 34 )^

Cost-effectiveness analysis poses many challenges and limitations due to the variety of specific treatment scenarios, drug, and hospital care prices required to calculate costs. Varying GDP among countries and the disparities among public and private systems also increase the difficulty of comparing published results. Analyzing our ICER values, we can observe that the main influencer is the drug’s price, while variations in the utilities, adverse events costs, and PPS cost had a low influence on the outcome.

## CONCLUSION

Our analysis showed that ado-trastuzumab emtansine is cost-effective for the treatment of residual disease after neoadjuvant treatment when compared to trastuzumab both in the USA and Brazil scenarios.
